# Influence of heat and moisture exchanger use on measurements performed with manovacuometer and respirometer in healthy adults

**DOI:** 10.1186/s40248-015-0037-9

**Published:** 2015-12-19

**Authors:** Jeanette Janaina Jaber Lucato, Thiago Marraccini Nogueira da Cunha, Sara Solange Oliveira Costa Rocha, Fernanda Maria Palmieri de Carvalho, Daniele Cristina Botega, Jamili Anbar Torquato, Ana Cristina Gimenes, Renato Fraga Righetti

**Affiliations:** 1 Department of Physiotherapy, Centro Universitário São Camilo, Avenida Nazaré, 1501, Ipiranga, 04263-200 Sao Paulo, SP Brazil; 2 Physiotherapist, São Paulo, SP Brazil; 3 Department of Rehabilitation, Hospital Sírio Libanês, São Paulo, SP Brazil

**Keywords:** Heat and moisture exchanger, Respirometer, Maximum inspiratory pressure, Maximum expiratory pressure, Manovacuometer

## Abstract

**Background:**

The use of evaluation tools such as the manovacuometer and respirometer is frequent and disinfection is usually limited to the external surfaces, which is insufficient and raises concerns because of the potential spread of infectious diseases. Hydrophobic heat and moisture exchangers (HME) are used in mechanical ventilation and have microbiological filters, which can possibly reduce contamination, increasing the safety of related procedures. It is unknown, however, if the addition of an exchanger affects the measurements obtained. Aim of this study was to verify if the use of an HME interferes in maximal inspiratory and expiratory pressures assessed using the manovacuometer and vital capacity evaluated using the respirometer in healthy adults.

**Methods:**

A controlled transversal trial was carried out. Twenty healthy young adults were included in the study. Vital capacity by respirometer and, maximal inspiratory pressure (MIP) and maximal expiratory pressure (MEP) were assessed with and without the use of HME.

**Results:**

No significant difference was found between the values pre and post HME use in vital capacity measurements: (3878.8 ± 202.2 mL vs. 3925.5 ± 206.0 mL, *p* = 0.116) and the respiratory muscle strength measurements: MIP (−99.0 ± 8.9 vs −95.5 ± 9.0 cm H_2_O, *p* = 0.149) and MEP (92.5 ± 7.5 vs 92.5 ± 7.7 cm H_2_O, *p* = 1.0) respectively.

**Conclusion:**

We conclude that the use of HME does not modify the lung volumes or respiratory muscle strength, and can be used in order to reduce the occurrence of pulmonary infection.

## Background

Respiratory muscle weakness may be present in patients with dyspnea, respiratory failure, malnourishment, or debility, in neuromuscular diseases such as Guillain-Barre syndrome, myasthenia gravis, amyotrophic lateral sclerosis, stroke, poliomyelitis, or in multisystem diseases such as polymyositis and sarcoidosis [1, 2]. Assessment of lung volumes and respiratory muscle strength has a low cost and can be easily obtained using the respirometer and manovacuometer , respectively [3].

These measurements are routinely used to monitor patients with acute conditions, at risk of rapid loss of diaphragm strength as well as to follow the progress of patients with chronic diseases and to detect muscle weakness in undiagnosed patients [1, 2, 4].

Vital capacity (VC), defined as the maximum amount of air that can be exhaled after a maximum inhalation, is an indispensable measurement for the diagnosis of pulmonary mechanical limitation as well as for assessment of pulmonary reexpansion therapy applied to patients after cardiac surgery. The normal value of the VC is from 65 to 75 mL/kg, however, there may be variations regarding ethnicity, age, gender, height and weight. It has been described that VC lower than 25 ml/Kg can predispose atelectasis, hypoxemia and inefficient cough, so the evaluation of pulmonary volumes and capacities is essential to characterize pulmonary mechanical limitation and restrictive pattern in some patients [5].

The manovacuometer, according to the manufacturer manual, is cleaned only externally, and it is believed that this aspect has a potential to increase the incidence of infections, since no device is used for filtering exhaled air and the air inspired by the.patient. This potential seems to be more relevant in intensive care unit (ICU) patients, since some of the risk factors for nosocomial pneumonia include long ICU stay, prior exposure to antibiotics and other drugs, invasive procedures and exposure to equipment, devices, hands, air, contaminated water and solutions [6].

Heat and moisture exchangers (HME) are disposable devices that are inserted between the endotracheal tube and the Y-piece of the mechanical ventilator [6–8]. HME recover heat and moisture during exhalation and return a portion of the heat and humidity during the following inspiration [9–13]; they are more efficient when used with low tidal volumes [13] and do not cause relevant increases in resistance [14]. There are 3 types of HME: hygroscopic, hydrophobic, and combined (hygroscopic-hydrophobic) [6]. Hydrophobic HME has a larger surface area, because of pleating of the material [10]; it has a substance covering the filter that prevents water exodus during exhalation, and serves as an efficient microbiologic filter as well [15].

The aim of the present study was to evaluate if the use of the hydrophobic HME interferes in the lung volumes, maximal inspiratory pressure (MIP) and maximal expiratory pressure (MEP) obtained using the respirometer and manovacuometry in healthy adults. The hypothesis is that using the HME coupled to these devices do not modify the values of muscle force and lung volumes, allowing them to be used in clinical practice.

## Methods

This was a prospective, randomized by envelope, descriptive study in a convenience sample comprising 20 healthy volunteers. The study included volunteers aged between 18 and 40 years old, both sexes, who agreed to provide written informed consent. The exclusion criteria were: presence of respiratory disease; recent abdominal or thoracic surgery; myopathies; acute middle ear problems; subjects refusing to provide written informed consent. All volunteers received information about their participation in the project and provided written informed consent, agreeing to take part in the study. This study was approved by the Committee on Ethics and Research of our institution (protocol number: 45/10).

### Respiratory muscle strength

**Fig. 1:**
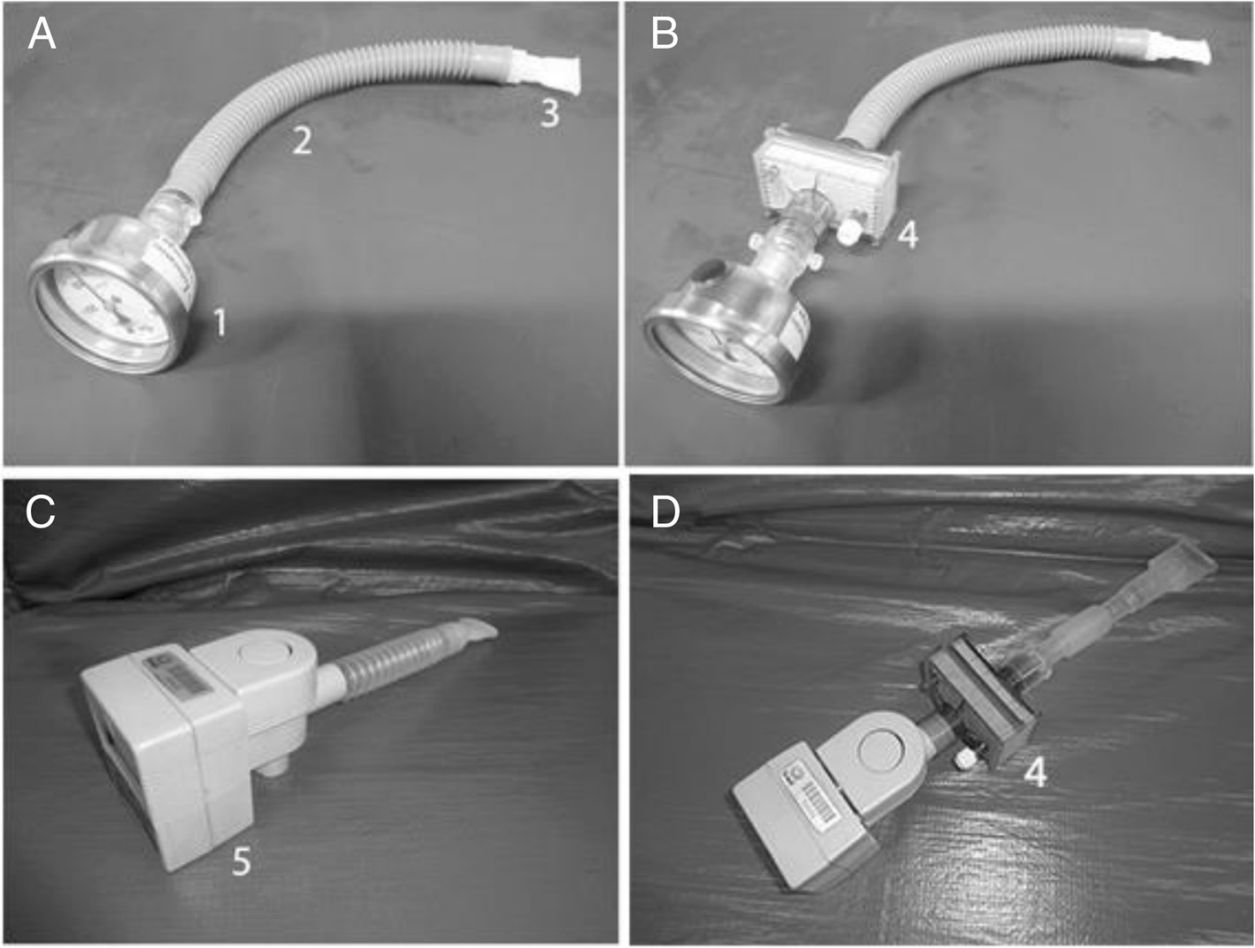
Photographs of the dispositives: **a** Manovacuometer used in a conventional way. **b** Manovacuometer and HME employed. **c** Respirometer used in a conventional way. **d** Respirometer and HME employed. Legend: (1) Manovacuometer; (2) Trachea; (3) Disposable mouthpiece; (4) HME and (5) Respirometer

The participants performed MIP and MEP using a manometer (Ger-AR® - São Paulo, Brazil) (Fig. 1a) and according to previously reported techniques [16]. The maneuvers were performed at rest. Attached to the manometer, a 1 mm hole in diameter was used to dissipate possible additional pressures caused by the facial muscles and the oropharynx [17]. To prevent air leakage, a nose clip was used and the patient was instructed to make proper adjustment of the lips around the flanged mouthpiece. To measure the MIP, the patient was instructed to perform a maximal inspiration, from the residual volume, and for the MEP, was guided to make a maximal expiration from total lung capacity. To minimize the use of accessory facial muscles, one of the investigators manually contained both cheeks during the MEP assessment. Participants were required to maintain pressure levels for at least one second and the best measure of three maneuvers was recorded with one-minute rest between each maneuver. Verbal encouragement was provided in all measurements.

The same evaluation procedure of MIP and MEP was repeated, but now using an HME (Model BB100MFS, Pall Corporation - Cornwall, United Kingdom) between the disposable flanged mouthpiece and the manovacuometer (Fig. 1b). This HME has hydrophobic characteristics and an ability to filter out 99 % of bacteria and viruses; it weighs 44 g, has a dead space of 90 mL and resistance of approximately 2.5 cm H_2_O/L/s [18].

The highest value recorded was used for analysis, and then compared with the corresponding value obtained without the HME for the same subject, both for MIP and MEP.

### Respirometer

Vital capacity was evaluated with a respirometer. The individuals were placed in a sitting position with thorax in a vertical way in approximately 90°. A respirometer (Model 00–295, Anesthesia Associates, Inc. - San Marcos, California, USA) was used with a 10 cm-long trachea, connected between the respirometer and a flanged mouthpiece (Fig. 1c). A nasal clip was used to avoid air escape by the nose. Then, the patients performed a quiet breathing for one minute to measure MV. Thereafter, the patients performed a deep inspiration until total pulmonary capacity followed by continuous and slow expiration until residual volume [5].

The same evaluation procedure of vital capacity was repeated, but now using an HME (Model BB100MFS, Pall Corporation - Cornwall, United Kingdom) between the disposable flanged mouthpiece and the respirometer (Fig. 1d). The highest value recorded was used for analysis, and then compared with the corresponding value obtained without the HME for the same subject.

### Data analysis

All statistical analysis was made using SigmaPlot version 11.0 (Systat Software, Inc. - San Jose, California, USA). All data represent means ± standard error (S.E.). Statistical significance of difference between groups was determined by Paired *t-*test. *P*< 0.05 was considered significant.

## Results

### Anthropometric characteristics

A total of twenty healthy subjects were selected, according to the above-cited inclusion criteria. Anthropometric characteristics and pulmonary function of the sample are described in Table 1.

**Table 1:** Anthropometric characteristics of healthy sujects

Characteristics	Data
Gender (M/F)	4/16
Age (years)	22.9 ± 2.8
Weight (kg)	58.8 ± 8.2
Height (m)	1.62 ± 0.08
BMI (kg/m^2^)	22.7 ± 1,7

### Inspiratory and expiratory muscle strength

**Fig. 2:**
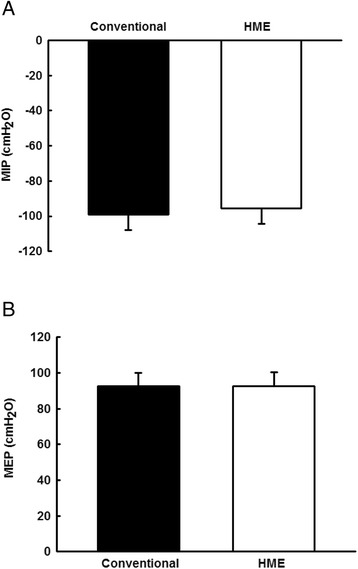
Graphic representation of respiratory pressures in both maneuvers. Vertical bar graphs representing the mean ± standard deviation of MIP **a** and MEP **b**, evaluated in a conventional manner or with the addition of an HME (*p* = 0.149 and 1.0 respectively; compared between maneuvers)

The values of the MIP evaluated in the conventional manner (MIP conventional: −99 ± 8.9 cm H_2_O) or associated with HME (MIP HME: −95.5 ± 9.0 cm H_2_O). There were no differences between conventional and HME maneuvers (*p* = 0.149; Fig. 2a).

The values of the MEP evaluated in the conventional manner (MEP conventional: 92.5 ± 7.5 cm H_2_O) or associated with HME (MEP HME: 92.5 ± 7.7 cm H_2_O). There were no differences between conventional and HME maneuvers (*p* = 1.0; Fig. 2b).

### Vital capacity

**Fig. 3:**
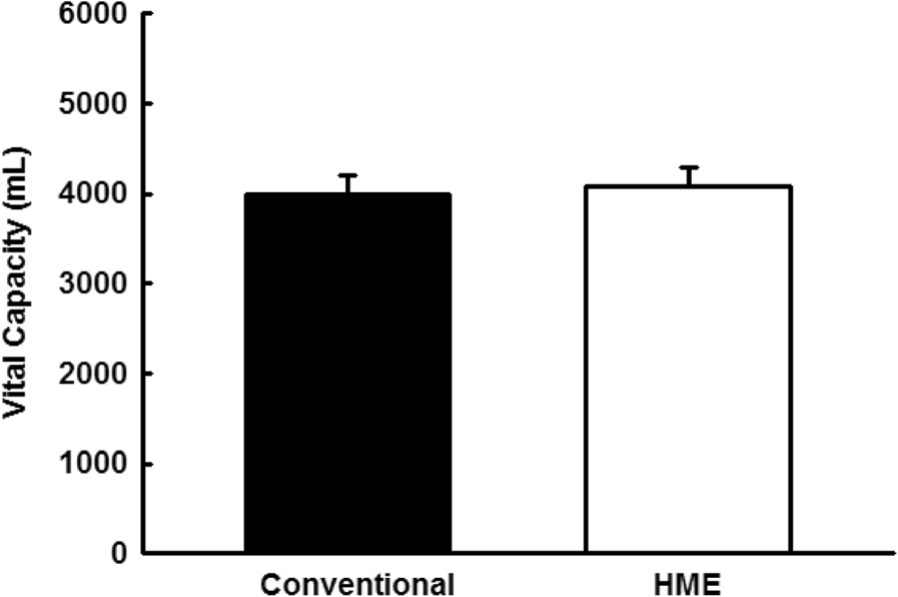
Graphic representation of vital capacity in both maneuvers. Vertical bar graphs representing the mean ± standard deviation, evaluated in a conventional manner or with the addition of an HME (*p* = 0.116; compared between maneuvers)

The values of the VC evaluated in the conventional manner (VC conventional: 3878.8 ± 202.2 mL) or associated with HME (VC HME: 3925.5 ± 206.0 mL). There were no differences between conventional and HME maneuvers (*p* = 0.116; Fig. 3).

## Discussion

We evaluated respiratory pressures and VC with and without the addition of an HME. Our results showed that, in our sample, there were no statistically significant differences between these two maneuvers, especially regarding MEP. This finding indicates that the above mentioned potential influence of the presence of the HME over respiratory pressures and VC is negligible. Our data point to the possibility of using HME in future studies when respiratory pressures and VC are assessed using the manovacuometer or respirometer.

The respiratory tract contains a nearly ideal environment for microbes, since they are warm, humid and dark. Several liters of air move into and from the lungs every minute, with the potential for carrying large numbers of microorganisms. Many of the patients encountered by the respiratory therapist have compromised host defenses against infection, and in the case of mechanically ventilated patient the respiratory therapist regularly bypasses these defenses. Therefore , a primary issue in respiratory care is to prevent the introduction of infection. Although the majority of nosocomial pneumonia cases arises from microaspiration, respiratory care equipment itself is a well-documented source of such infection [19].

Efficient bacterial filtration, by means of using a hydrophobic HME, can be important, especially in immunocompromised patients infected or in the ICU and can help protect the patient from cross bacterial contamination [20].

We chose to use the hydrophobic HME BB100MFS due to a previous study that linked the use of HME with decreasing nosocomial infection in patients on mechanical ventilation [18]. However, despite showing a reduction of infection, there are no reports on how its use can influence the values of respiratory pressures or VC.

Theoretically, as the filter volume is relatively large, the aspirated gas volume would change the basal lung volume or the conformation of the diaphragm during MIP measurements, which in turn would affect the accuracy of measurements. An increase in respiratory workload due to filter resistance could lead to the underestimation of MEP and MIP because of various reasons such as respiratory muscle fatigue, but in the present study the 90 mL of the death space and resistance of approximately 2.5 cm H_2_O/L/s of the HME doesn’t affect the lung volumes and respiratory pressures [1, 2, 14].

The determinants of resistive pressure include not only the airway, but also the entire breathing circuit used including HME [21]. The resistance to gas flow along an HME increases with the density of the material, with increased flow and the duration of use [15] and [22] in the mechanical ventilation, as the usage time of the HME water will be absorbed by the filter and the resistance increases according to the prolonged use [23]. In this study, we used the HME only for measures of respiratory muscle strength and vital capacity in patients breathing spontaneously, excluding the possibility of a prolonged use of HME and a significant interference resistance to airflow generated by the patient.

Due to its internal volume, not negligible, the HME increases the dead space of the respiratory circuits in mechanically ventilated patients [7, 24]. In a comparison between two HMEs configuration and with identical chemical composition but with different sizes it was concluded that it was preferable to use the HME with smaller internal volume for spontaneously breathing patients. The larger HME increased patient effort, but with no sign of respiratory distress, while the smaller HME did not add a detectable load [25]. The use of HME determines increased dead space in an amount equal to its internal volume. The filters are usually made of pleated membrane, which result in increased volume of the device. In general, the HMEs without the filter function have a smaller dead space [26]. There are no studies evaluating the effects of HME over lung volumes and respiratory muscle strength in patients breathing spontaneously.

In this study, in patients breathing spontaneously, the volume of dead space related to the addition of an HME was not able to change the values of VC and respiratory muscle strength. However, the present study represents an initial effort in order to assess these questions, as the subjects accrued were healthy young adults. Further studies, focusing on patients with low body weight and/or disturbed basal pulmonary function, will add pertinent data to this topic, trying to verify if the addition of the filter leads to relevant impact in this subgroup.

## Conclusion

There was no difference between the evaluation of respiratory pressures and vital capacity using the manovacuometer and respirometer with or without an HME. Therefore, the addition of a hydrophobic HME, with microbial filtration capacity, might be a simple solution to reduce the potential of contamination of manovacuometer and respirometer.

## Abbreviations

HME: Heat and moisture exchanger
VC: Vital capacity
ICU: Intensive care unit
HME: Heat and moisture exchanger
MIP: Maximal inspiratory pressure
MEP: Maximal expiratory pressure

## References

[1] DePalo VA, McCool FD (2002). Respiratory muscle evaluation of the patient with neuromuscular disease. Semin Respir Crit Care Med.

[2] Baydur A, Alsalek M, Louie SG, Sharma OP (2001). Respiratory muscle strength, lung function, and dyspnea in patients with sarcoidosis. Chest.

[3] American Thoracic Society/European Respiratory Society. ATS/ERS Statement on respiratory muscle testing. Am J Respir Crit Care Med. 2002; 166: 518–624. 10.1164/rccm.166.4.518 12186831

[4] Evans JA, Whitelaw WA (2009). The assessment of maximal respiratory mouth pressures in adults. Respir Care.

[5] Pinheiro AC, Novais MC, Neto MG, Rodrigues MV, de Souza Rodrigues E, Aras R (2011). Estimation of lung vital capacity before and after coronary artery bypass grafting surgery: a comparison of incentive spirometer and ventilometry. J Cardiothorac Surg.

[6] Craven ED, Steger KA, La Force FM, Bennett JV, Brachman PS (1998). Pneumonia. Hospital infections.

[7] Ricard JD, Le Miere E, Markowicz P, Lasry S, Saumon G, Djedaini K (2000). Efficiency and safety of mechanical ventilation with a heat and moisture exchanger changed only once a week. Am J Respir Crit Care Med.

[8] Martin C, Papazian L, Perrin G, Bantz P, Gouin F (1992). Performance evaluation of three vaporizing humidifiers and two heat and moisture exchangers in patients with minute ventilation > 10 L/min. Chest.

[9] Markowicz P, Ricard JD, Dreyfuss D, Mier L, Brun P, Coste F (2000). Safety, efficacy, and cost-effectiveness of mechanical ventilation with humidifying filters changed every 48 hours: a prospective, randomized study. Crit Care Med.

[10] Holt TO, Barnes TA (1994). Aerosol generators and humidifiers. Core textbook of respiratory care practice.

[11] Hess DR, Branson RD, Branson RD, Hess DR, Chatburn RL (1999). Humidification. Respiratory care equipment.

[12] Thomachot L, Viviand X, Boyadjiev I, Vialet R, Martin C (2002). The combination of a heat and moisture exchanger and a Booster: a clinical and bacteriological evaluation over 96 h. Intensive Care Med.

[13] Hurni JM, Feihl F, Lazor R, Leuenberger P, Perret C (1997). Safety of combined heat and moisture exchanger filters in long-term mechanical ventilation. Chest.

[14] Lucato JJ, Adams AB, Souza R, Torquato JA, Carvalho CR, Marini JJ (2009). Evaluating humidity recovery efficiency of currently available heat and moisture exchangers: a respiratory system model study. Clinics (Sao Paulo).

[15] Lucato JJ, Tucci MR, Schettino GP, Adams AB, Fu C, Forti G (2005). Evaluation of resistance in 8 different heat-and-moisture exchangers: effects of saturation and flow rate/profile. Respir Care.

[16] Neder JA, Andreoni S, Lerario MC, Nery LE (1999). Reference values for lung function tests. II. Maximal respiratory pressures and voluntary ventilation. Braz J Med Biol Res.

[17] Fregonezzi GAF, Resqueti VR, Guell R, Pradas J, Casan P (2005). Effects of 8 week, Interval-Bases inspiratory muscle training and breathing retraining in patients with generalized myasthenia gravis. Chest.

[18] Thomachot L, Viviand X, Arnaud S, Boisson C, Martin CD (1998). Comparing two heat and moisture exchangers, one hydrophobic and one hygroscopic, on humidifying efficacy and the rate of nosocomial pneumonia. Chest.

[19] Carter C, Stone MK, Hess DR, MacIntyre NR, Mishoe SC, Galvin WF, Adams AB, Saposnick AB (2002). Respiratory microbiology, infection, and infection control. Respiratory care: principles & practice.

[20] Bench S. Humidification in the long-term ventilated patient; a systematic review. Intensive Crit Care Nurs. 2003; 19(2):75-84. 10.1016/s0964-3397(03)00024-7 12706733

[21] Manthous CA, Schmidt GA (1994). Resistive pressure of a condenser humidifier in mechanically ventilated patients. Crit Care Med.

[22] Ploysongsang Y, Branson R, Rashkin MC, Hurst JM (1988). Pressure flow characteristics of commonly used heat-moisture exchangers. Am Rev Respir Dis.

[23] Nishimura M, Nishijima MK, Okada T, Taenaka N, Yoshiya I (1990). Comparison of flow-resistive work load due to humidifying devices. Chest.

[24] Iotti GA, Olivei MC, Palo A, Galbusera C, Veronesi R, Comelli A (1997). Unfavorable mechanical effects of heat and moisture exchangers in ventilated patients. Intensive Care Med.

[25] Natalini G, Bardini P, Latronico N, Candiani A (1994). Impact of heat and moisture exchangers on ventilatory pattern and respiratory mechanics in spontaneously breathing patients. Monaldi Arch Chest Dis.

[26] Iotti GA, Olivei MC, Braschi A (1999). Mechanical effects of heat-moisture exchangers in ventilated patients. Crit Care.

